# Deficiency of GPR10 and NPFFR2 receptors leads to sex-specific prediabetic syndrome and late-onset obesity in mice

**DOI:** 10.1042/BSR20241103

**Published:** 2024-10-23

**Authors:** Alena Morgan, Nivasini Shekhar, Veronika Strnadová, Zdenko Pirník, Eliška Haasová, Jan Kopecký, Andrea Pačesová, Blanka Železná, Jaroslav Kuneš, Kristina Bardová, Lenka Maletínská

**Affiliations:** 1 Institute of Organic Chemistry and Biochemistry of the Czech Academy of Sciences, 166 10 Prague, Czech Republic; 2 Institute of Physiology of the Czech Academy of Sciences, 142 00 Prague, Czech Republic; 3 Department of Physiology, Faculty of Science, Charles University in Prague, 128 44 Prague, Czech Republic; 4 Biomedical Research Center, Slovak Academy of Sciences, 845 05 Bratislava, Slovak Republic; 5 Institute of Physiology, Faculty of Medicine, Comenius University in Bratislava, 813 72 Bratislava, Slovak Republic

**Keywords:** double KO mice, GPR10/NPFFR2-deficient mice, impaired glucose utilization, insulin resistance, obesity

## Abstract

GPR10 and neuropeptide FF receptor 2 (NPFFR2) play important role in the regulation of food intake and energy homeostasis. Understanding the interaction between these receptors and their specific ligands, such as prolactin-releasing peptide, is essential for developing stable peptide analogs with potential for treating obesity. By breeding and characterizing double knockout (dKO) mice fed standard or high-fat diet (HFD), we provide insights into the metabolic regulation associated with the GPR10 and NPFFR2 deficiency. Both WT and dKO mice were subjected to behavioral tests and an oral glucose tolerance test. Moreover, dual-energy X-ray absorptiometry (DEXA) followed by indirect calorimetry were performed to characterize dKO mice. dKO mice of both sexes, when exposed to an HFD, showed reduced glucose tolerance, hyperinsulinemia, and insulin resistance compared with controls. Moreover, they displayed increased liver weight with worsened hepatic steatosis. Mice displayed significantly increased body weight, which was more pronounced in dKO males and caused by higher caloric intake on a standard diet, while dKO females displayed obesity characterized by increased white adipose tissue and enhanced hepatic lipid accumulation on an HFD. Moreover, dKO females exhibited anxiety-like behavior in the open field test. dKO mice on a standard diet had a lower respiratory quotient, with no significant changes in energy expenditure. These results provide insights into alterations associated with disrupted GPR10 and NPFFR2 signaling, contributing to the development of potential anti-obesity treatment.

## Introduction

Prolactin-releasing peptide (PrRP) is a member of the RF-amide neuropeptide family that regulates multiple physiological processes and feeding behavior (reviewed in [1,2]). Previous studies have shown that intracerebroventricular (i.c.v.) administration of PrRP reduced food intake and increased energy expenditure (EE) [3,4]. Mice with a disrupted gene encoding *Prrp* showed adult-onset obesity, high body fat levels, impaired glucose tolerance and increased insulin resistance [5], and showed higher concentrations of blood glucose and corticosterone compared with wild-type (WT) mice after restraint stress [6].

PrRP is an endogenous agonist of G-protein coupled receptor 10 (GPR10), which is found in various brain regions responsible for appetite including energy regulation, such as the paraventricular (PVN), ventromedial, and dorsomedial hypothalamic nucleus (DMN), and the nucleus tractus solitarius (NTS) [7,8]. GPR10 is highly expressed in hypothalamic PVN, DMN and ventromedial hypothalamus (VMH) as well as in the pituitary gland and medulla oblongata [9]. Additionally, in peripheral tissues, it exhibits high expression levels in the rat adrenal medulla [10] and testis [11]. PrRP has also been identified as a potent agonist of the neuropeptide FF receptor 2 (NPFFR2), which is present in the brain areas involved in regulating feeding behavior and energy balance such as amygdala, DMN, VMH, lateral hypothalamic area (LHA), PVN, and medulla oblongata, similar to GPR10 [12–14]. In periphery, *NPFFR2* mRNA is highly expressed in the placenta and adipose tissue in rats and humans [15], but at lower levels in the heart, kidney, and lung in rats [16].

GPR10 deficiency led to a higher body weight due to increased adipose tissue mass, resulting in elevated plasma leptin levels. GPR10 knockout (KO) mice also exhibited elevated insulin levels and impaired glucose tolerance, suggesting increased susceptibility to diabetes [17–19]. Zhang et al. demonstrated that NPFFR2 KO mice exhibited decreased diet-induced adaptive thermogenesis [20]. Additionally, our previous study showed that NPFFR2 KO mice had a lean phenotype but exhibited significantly impaired glucose tolerance, particularly when fed a high-fat diet (HFD) [21]. Previous studies found that i.c.v. administration of PrRP positively affected the hypothalamic–pituitary–adrenal axis (HPA) by increasing adrenocorticotropin (ACTH) [22] and corticosterone levels [23] in rats.

While natural PrRP affects food intake only when administered centrally, lipidized PrRP effectively reduces food intake by central effects, even after peripheral administration, due to prolonged stability, preserved GPR10 binding and increased binding to NPFFR2 [24]. The activation of both GPR10 and NPFFR2 receptors was found to be essential for the anti-obesity effects of PrRP [24–26]. In agreement, chronic treatment with lipidized PrRP analogs effectively suppresses appetite, reduces body weight, and improves glucose tolerance in rodents with diet-induced obesity (DIO) [25–27].

In the present study, for the first time, we aimed to examine the importance of GPR10 and NPFFR2 receptors in the regulation of energy metabolism and obesity development using GPR10/NPFFR2 KO (dKO) mice. This study is particularly relevant considering the potential use of lipidized analogs for the treatment of obesity and type 2 diabetes mellitus.

## Methods

### Mice and animal care

All animal experiments were approved by the Committee for Experiments with Laboratory Animals of the Czech Academy of Sciences of the Czech Republic and performed with ethical standards according to the ethical guidelines of the EU (86/609/EU) and the Act of the Czech Republic law 246/1992 (project No. 96/2020). The study and the methodology were designed in accordance with the ARRIVE guidelines. C57BL/6J *Gpr10*^−/−^ homozygous mice (previously described in [19]) were crossbred with C57BL/6N *Npffr2*^−/−^ homozygous mice (previously described in [21]) to generate *Gpr10*^−/−^ and *Npffr2*^−/−^ mice of both sexes (Supplementary Figure S1). These mice were bred in the Czech Centre for Phenogenomics, Institute of Molecular Genetics of the Czech Academy of Sciences, Czech Republic. The correct genome editing was verified through PCR amplification in mice using primers designed for Npffr2 (Npffr2-F: 5′-CTTCCGATGTCCACCTGTCT-3′; Npffr2-R: 5′-GGTGGTACCAGCGAAGTGAT-3′) and Gpr10 (Gpr10-F: 5′-GCGATTGTGGTGAGTGTGAACACCG-3; Gpr10-R: 5′-AGAGCAGGCATTTATCTAATCCCACC-3′). Control WT mice were obtained through the crossbreeding of C57BL/6J and C57BL/6N strains from the same source.

Mice were housed in groups of two in the animal facility under standard conditions (12/12h light-dark cycle, temperature 23 ± 2°C) and had free access to water and a standard chow diet (STD; Ssniff R/M-H diet; 8% kcal from fat, 21% kcal from protein, and 71% kcal from carbohydrate; Ssniff Spezialdieten GmbH, Soest, Germany).

### Experimental design

**Figure 1:**
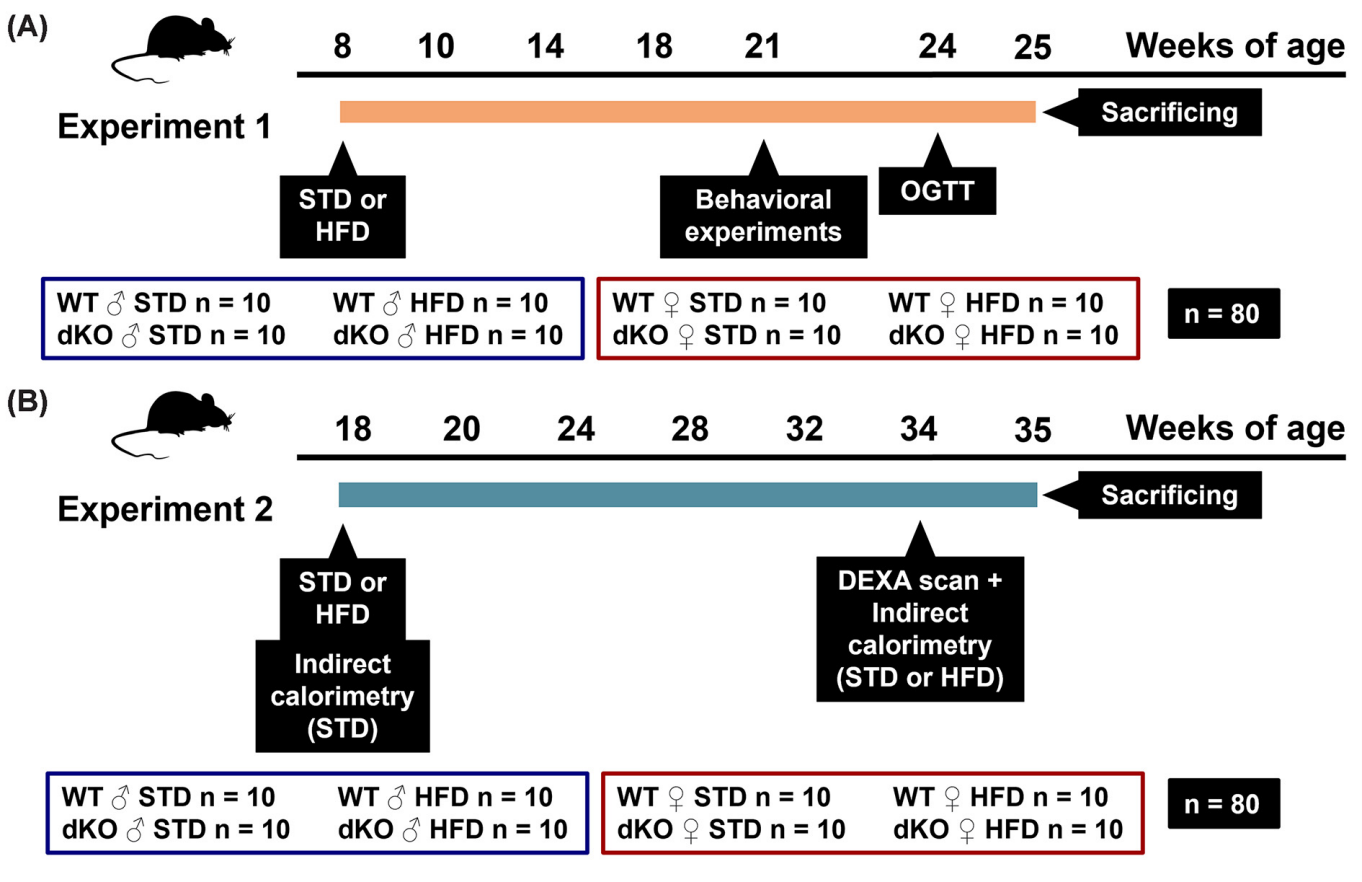
Experimental design In Experiment 1 (**A**), dKO and WT male and female mice were exposed to either a STD or a HFD from the age of 8 weeks (*n* = 10/group). At the age of 21 weeks, mice were subjected to behavioral tests, and at the age of 24 weeks, they were subjected to an OGTT. Mice were sacrificed at the age of 25 weeks. In Experiment 2 (**B**), WT and dKO mice were therefore exposed to STD or HFD (*n* = 10/group) from the age of 18 weeks. DEXA scanning and indirect calorimetry was performed on STD-fed mice. DEXA scanning was repeated in 34-week-old mice after 16 weeks of dietary intervention, followed by indirect calorimetry. dKO double knock-out, DEXA, dual-energy X-ray absorptiometry; HFD, high-fat diet; OGTT, oral glucose tolerance test; STD, standard diet.

In Experiment 1 (for the experimental design, see Figure 1A), dKO and WT mice (total, *n*=80) were exposed to either a standard diet (STD) (male, *n* = 10/group and female, *n* = 10/group) or HFD (13% protein kcal, 60% fat kcal and 27% carbohydrate; as previously described in [24]) (male, *n* = 10/group and female, *n* = 10/group) from the age of 8 weeks, and body weight and spontaneous food intake were monitored weekly.

At the age of 21 weeks, mice were subjected to behavioral and pain perception tests, and at the age of 24 weeks, they were subjected to an oral glucose tolerance test (OGTT). Mice fed *ad libitum* were sacrificed at the age of 25 weeks under intraperitoneal pentobarbital anesthesia (80 mg/kg of body weight, Sigma-Aldrich, St. Louis, MO, U.S.A.) and intracardially perfused with heparinized saline (20 U/ml).

Samples of subcutaneous and gonadal white adipose tissue (scWAT and gWAT, respectively), the liver and hypothalamus and free-fed plasma of all mice were collected and stored at −80°C until use. For liver histology, the caudate lobe was utilized. Various analyses were conducted, including plasma analysis of biochemical parameters, liver histology, Western blots (WB), and quantitative real-time PCR (qRT-PCR).

In Experiment 2, building upon the significant impact of the HFD observed in both WT and dKO mice (total, *n*=80) from 16 weeks of age in Experiment 1, we proceeded to examine dKO mice in a distinct cohort at an older age, employing a modified experimental design (Figure 1B).

WT and dKO mice were therefore exposed to either STD (male, *n* = 10/group and female, *n* = 10/group) or HFD (male, *n* = 10/group and female, *n* = 10/group) from the age of 18 weeks, and body weight was monitored every other week; and dual-energy X-ray absorptiometry (DEXA) scanning and indirect calorimetry was performed on STD-fed mice to reveal the effects of genotype. DEXA scanning was repeated in 34-week-old mice after 16 weeks of dietary intervention followed the next day by indirect calorimetry. Mice were killed by a decapitation a week later.

### Oral glucose tolerance test (OGTT)

After 6 h of fasting, mice at the age of 24 weeks (Experiment 1) were subjected to an OGTT. Before the experiment, fasting blood glucose was measured, and blood samples were collected from the tail veins for subsequent analysis of fasting plasma. By gavage, glucose was given orally at a dose of 2 g/kg of body weight (dissolved in distilled water), and the concentration of blood glucose was measured at 15, 30, 60, 90, 120, and 180 min using a glucometer (LifeScan, Inc., Milpitas, CA, U.S.A.). Insulin resistance was assessed by calculating the Homeostatic Model Assessment for Insulin Resistance (HOMA-IR) index according to the following formula: HOMA-IR = (fasting plasma insulin concentration [mU/L] × fasting plasma glucose [mmol/L]) / 22.5 [28].

### Behavioral test and the pain response test

We conducted the open field (OF) test to evaluate exploration, anxiety, and locomotor activity and a hot plate (HP) system to evaluate pain perception (Experiment 1). The behavioral experiments followed a previously published procedure [21].

Mice were individually placed in the same spot within a square testing zone (50 × 50 cm), and their locomotor activity was recorded for 10 min in the OF test. Subsequently, the total track length, time spent in the central zone, and frequency of entries into the central zone were quantified. To evaluate the records, EthoVision XT (Noldus, Wageningen, Netherlands) was used.

Pain perception was evaluated using a hot plate (HP) system (TSE Systems, Bad Homburg, Germany) set at 53°C with a cut-off time of 60 s. The time from placing the animal on the hot plate surface to the onset of nociceptive behavior (front paw licking) was recorded. After every trial session, the mice were immediately removed from the plate and returned to the cage.

### Blood analysis

The concentration of insulin was measured in fasting plasma (Experiment 1) collected before OGTT using radioimmunoassay (#SRI-13K; Merck Millipore, Burlington, MA, U.S.A.).

Colorimetric assays were used to determine the levels of cholesterol (#BLT00036), triacylglycerol (TAG, #BLT00059; Erba Lachema, Brno, Czech Republic) in fasting plasma (Experiment 1). The manufacturer’s instructions were followed for all measurements.

Plasma from *ad libitum*-fed anesthetized mice (Experiment 1) was collected to measure leptin (#EZML-82BK; Merck Millipore), total (#EZRGRA-91K) and active ghrelin (#EZRGRA-90K; Merck Millipore), and fibroblast growth factor 21 (FGF21; #EZRMFGF21; Merck Millipore) levels.

Plasma from *ad libitum*-fed anesthetized mice (Experiment 2) was collected to measure prolactin (PRL; #ab100736; Abcam, Littleton, Cambridge, U.K.), PrRP (#S-1407; BMA Biomedicals, Augst, Switzerland), and NPFF (#abx154455; Abbexa Ltd, Cambridge, U.K.) levels.

### Liver histology and morphometric analysis

The caudate lobes of liver from Experiment 1 (*n*=5) were carefully removed and fixed in 4% paraformaldehyde (PFA) in 0.1 M phosphate buffer at pH 7.4 for 24 h. Subsequently, they were transferred to 70% ethanol at 4°C prior to tissue processing. Livers were prepared in a Leica ASP200S tissue processor (Leica Biosystems, Buffalo Grove, IL, U.S.A.) and embedded in paraffin blocks.

The livers were cut on a Leica RM2255 microtome (Leica Biosystems,) into 5-μm-thick liver sections and processed as previously described [19]. Briefly, slices were deparaffinized in xylene and then rehydrated using a gradient of ethanol concentrations. They were stained for 2 min using Weigert’s iron hematoxylin solution (#HT1079-1SET; Merck Millipore), rinsed in tap water, and further stained for 1 min in 0.5% eosin Y stain C.I. 45380 (#R70892; Carl Roth GmbH + Co., Karlsruhe, Germany). After staining, the samples were rinsed in tap water, dehydrated, and finally covered with DPX mounting medium (#06522; MilliporeSigma, Burlington, MA, U.S.A.). Representative photomicrographs were taken with an Olympus IX83 microscope (Olympus Europa SE & Co. KG, Hamburg, Germany).

### RNA extraction and quantitative RT-PCR

Total tissue RNA was isolated, and gene expression was evaluated as described [29] from samples from Experiment 1 (*n*=5). Briefly, samples were homogenized, and total RNA was extracted. Reverse transcription to synthesize first-strand cDNA was performed using the High-Capacity cDNA Reverse Transcription Kit (Applied Biosystems, Foster City, CA, U.S.A.) with random primers, following the manufacturer’s instructions. The mRNA expression levels of genes of interest (Supplementary Table S1) was determined using a LightCycler 480 instrument (Roche Diagnostics, Mannheim, Germany).

Data were normalized to the geometric mean signal of reference genes (liver geomean of *M2B* and *Gapdh*; BAT *Gapdh*; hypothalamus *Ywhaz*) to compensate for variations in input RNA amounts and the efficiency of reverse transcription. Background gene expression levels were defined by a mean *C*p value (the cycle at which the fluorescence of a sample rises above the background fluorescence) lower than 30. Gene abbreviations and an overview of primer sequences are provided in Supplementary Table S1. All mRNA probes were purchased from Generi Biotech s.r.o. (Hradec Králové, Czech Republic).

### Hormonal panel and vaginal smears

A bead-based multiplex assay using the Luminex® xMAP® (#MSHMAG-21K; Merck Millipore) method was used to measure the levels of sex hormones and corticosterone from collected tail plasma from WT (*n*=20) and dKO (*n*=20) mice of both sexes fed *ad libitum* with STD at the age of 15 weeks. To evaluate murine estrus stages in females, vaginal smears were collected and stained with 0.1% Crystal Violet, as previously described [30]. Photomicrographs were obtained using an Olympus IX83 microscope.

### DEXA scanner

Using a DEXA scanner (InAlyser, Medicors, Korea) under light isoflurane anesthesia, fat mass and lean body mass was evaluated in 18- and 34-week-old WT and dKO mice (Experiment 2) of both sexes on STD and HFD.

### Indirect calorimetry

Measurements were performed using the 16-chamber system (TSE, Bad Homburg, Germany), which also allows detailed food intake and physical activity recording. Oxygen consumption (*V*O_2_) and carbon dioxide production (*V*CO_2_) were recorded every 5 min; the respiratory quotient (RQ; i.e., *V*CO_2_/*V*O_2_ ratio) marked substrate partitioning. EE was calculated using the following equation: EE (kcal/h) = 3.9 × *V*O_2_ (l/h) + 1.1 × *V*CO_2_ (l/h). Energy metabolism of 18- and 34-week-old mice was assessed for 48 h using indirect calorimetry with 24-h-long acclimatization period; and the last 24 h were used for analysis. Data were analyzed with respect to the light/dark cycle in 12 h periods.

### Statistical analysis

Data are presented as the mean ± SEM. Statistical analyses were performed using GraphPad Prism 8 software (GraphPad Software, Inc., San Diego, CA, U.S.A.). Differences were considered significant at *P*<0.05. Two-way ANOVA with the Bonferroni post hoc test was used to evaluate the difference between WT or dKO mice on either STD or HFD, and unpaired Student’s *t* test was used for analyses of the hormonal panel between WT and dKO mice on STD.

Differences in EE were evaluated by multiple linear regression using genotype, sex, lean body mass and fat mass as covariates [31]. Expression of results of EE measurement is assumed to be correct only if ANCOVA or multilinear regression is employed compared with misleading representation of results per animal, body weight or lean body mass [32,33].

## Results

### dKO mice showed significantly increased body weight in males fed STD and females fed HFD

**Figure 2:**
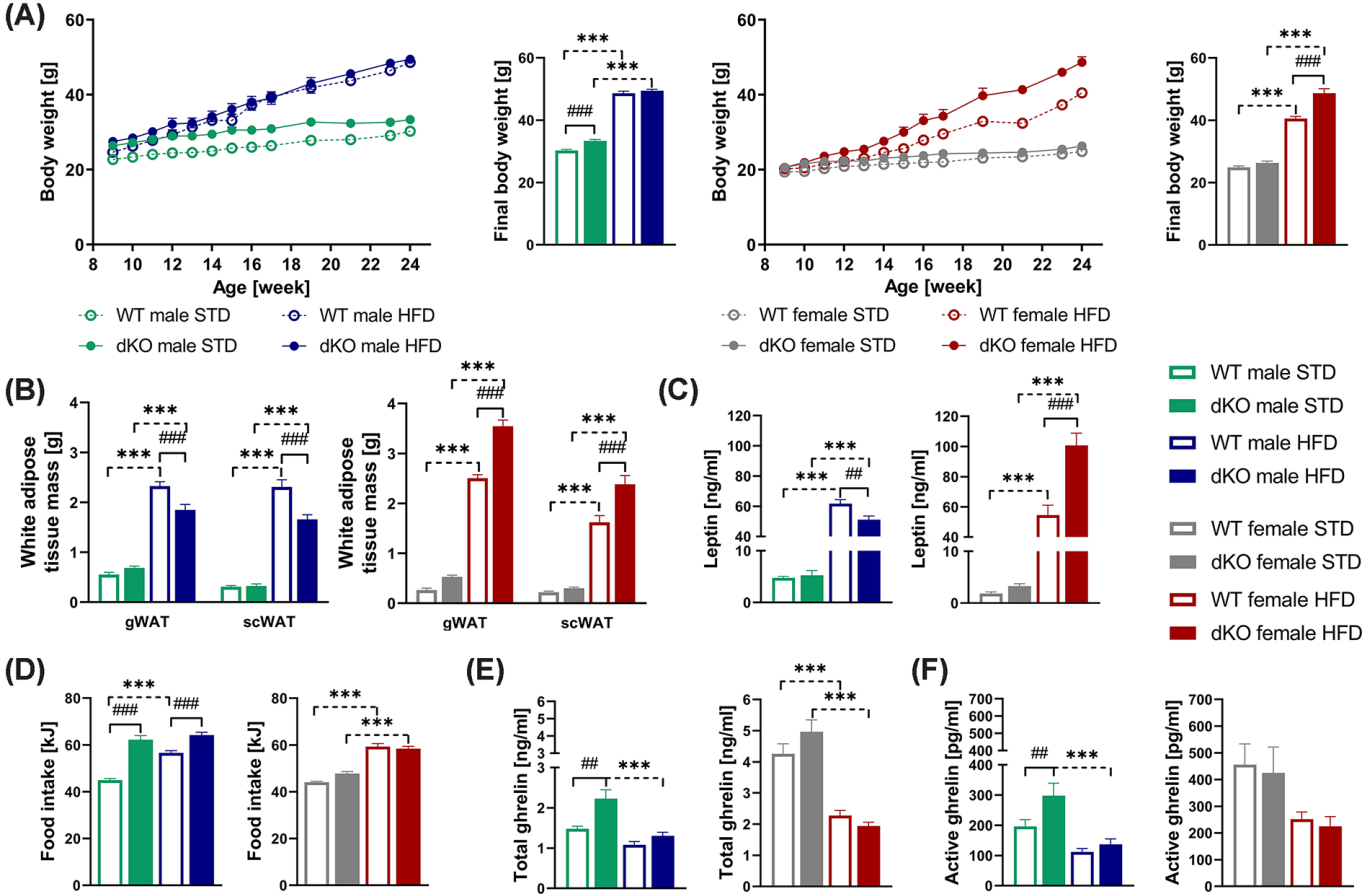
Morphometric and metabolic parameters of dKO mice on body weight, adipose tissue mass, and caloric intake Time course of body weight gain and final body weight (**A**) in WT and dKO mice on STD or HFD (*n*=9–10), weights of dissected gonadal white adipose tissue (gWAT) and subcutaneous white adipose tissue (scWAT) in males (*n*=10) and females (*n*=9–10) (**B**) at the age of 25 weeks. Plasma leptin levels (*n*=9–10) in males and females (**C**), and an average daily caloric intake in males and females (**D**). Total ghrelin levels (**E**) and active ghrelin levels (**F**) from plasma of WT and dKO free-fed mice on either STD or HFD (*n*=8–10). All data are expressed as the mean ± SEM and evaluated by two-way ANOVA with Bonferroni post hoc test. ##*P*<0.01, and ###*P*<0.001 for WT vs. dKO mice on the same diet and ***P<0.001 for STD vs. HFD mice of the same genotype.

To investigate the importance of GPR10 and NPFFR2 on energy balance, we placed 8-week-old male and female dKO mice and their WT controls on either a STD or HFD for 17 weeks (Experiment 1). We regularly monitored body weight and weighed dissected tissues at the age of 25 weeks. Male dKO mice fed STD exhibited significantly higher body weights than WT controls (Figure 2A,B). Interestingly, dKO males fed HFD exhibited significant reductions in gWAT and scWAT mass (Figure 2B) and a significant decrease in plasma leptin levels compared with WT males on HFD (Figure 2C).

The growth curve and final body weight of dKO females fed STD were comparable to those of WT controls. However, the body weights of dKO females fed HFD were significantly higher than those of WT controls (Figure 2A,B). Also, significant increases in gWAT and scWAT mass and plasma leptin levels were observed in dKO females fed HFD compared with WT controls (Figure 2B,C).

The daily caloric intake was significantly increased in dKO males fed STD (Figure 2D), and the total (Figure 2E) and active ghrelin plasma levels were increased (Figure 2F) in a free-fed state compared with WT controls. No significant changes in ghrelin levels and food intake between genotypes, only between diets were observed in females (Figure 2D–F).

### dKO mice fed HFD displayed increased glucose intolerance and insulin resistance

**Figure 3:**
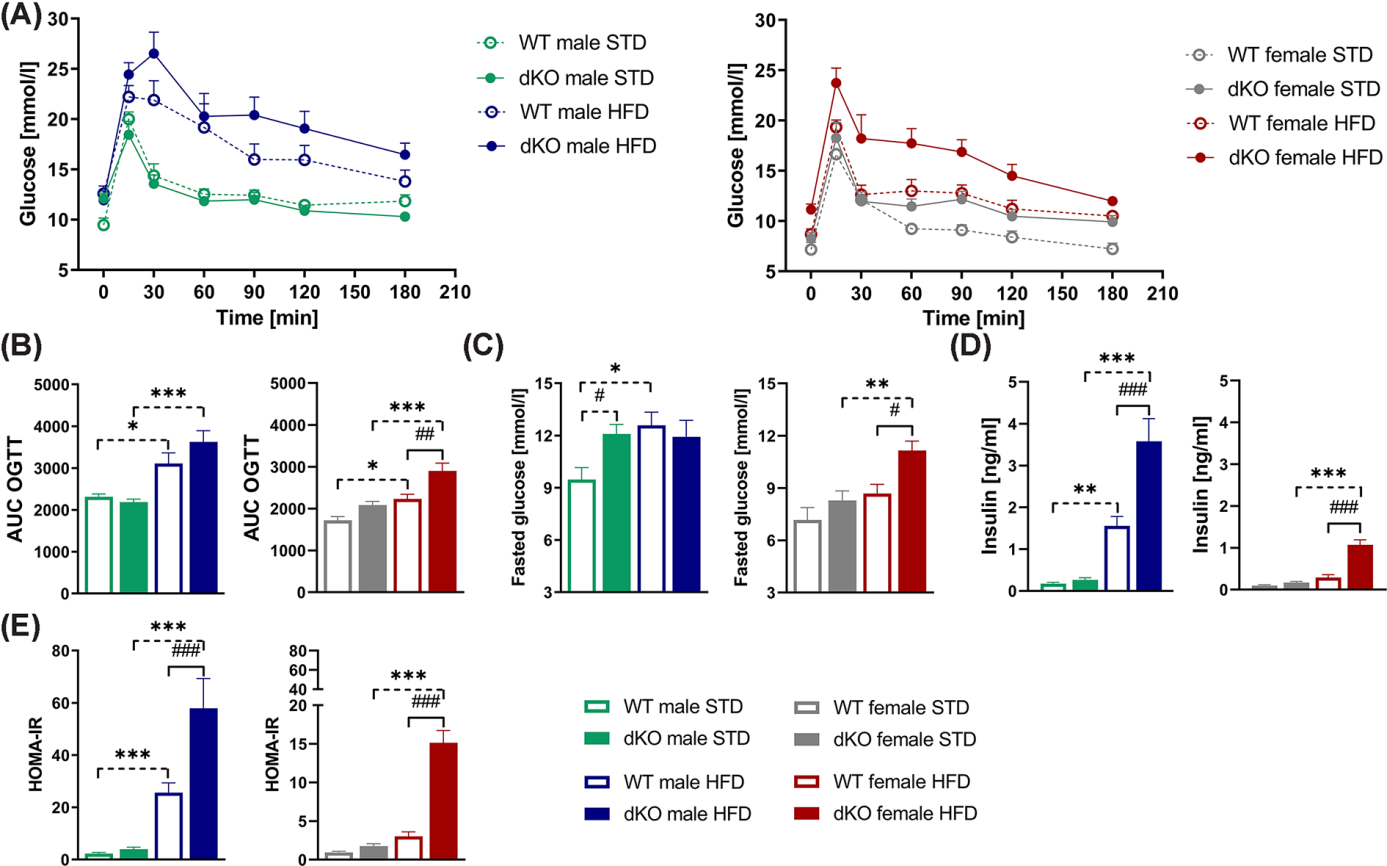
Impact of GPR10 and NPFFR2 deletion on glucose tolerance, and insulin levels Glucose excursions after glucose gavage (2 g/kg of body weight) of WT and dKO mice fed either STD or HFD (**A**). Area under the curve (AUC) from OGTT for males and females (**B**). Fasting glucose levels (*n*=10) in males and females (**C**). Insulin levels (*n*=6–10) in males and females (**D**) and indicator of insulin resistance HOMA-IR in males and females (**E**). Data are expressed as the mean ± SEM and were evaluated by two-way ANOVA with Bonferroni post hoc test. #*P*<0.05, ##*P*<0.01, and ###*P*<0.001 for WT vs. dKO mice on the same diet and *P<0.05,***P<0.01 and ***P<0.001 for STD vs. HFD mice of the same genotype.

To investigate the metabolic phenotyping of dKO mice under various dietary conditions, we assessed glucose and insulin levels, as well as insulin resistance markers, in both male and female mice. Twenty-four-week-old dKO males showed no significant differences in glucose levels or area under the curve (AUC) between genotypes on STD or HFD (Figure 3A,B). But after 6 h of fasting, dKO males fed STD showed significantly increased glucose levels compared with their WT controls (Figure 3C).

On the other hand, dKO females on HFD had significantly elevated blood glucose levels after glucose gavage and a significantly higher AUC than WT mice (Figure 3A,B). Moreover, dKO females showed significantly increased fasting glucose levels only when fed HFD compared with their WT controls (Figure 3C).

Both dKO males and females on HFD had significantly increased insulin levels in fasting plasma (Figure 3D), and they exhibited significantly higher HOMA-IR values than their respective WT controls (Figure 3E).

### Female dKO mice showed increased plasma cholesterol and FGF21 levels and liver steatosis

**Figure 4:**
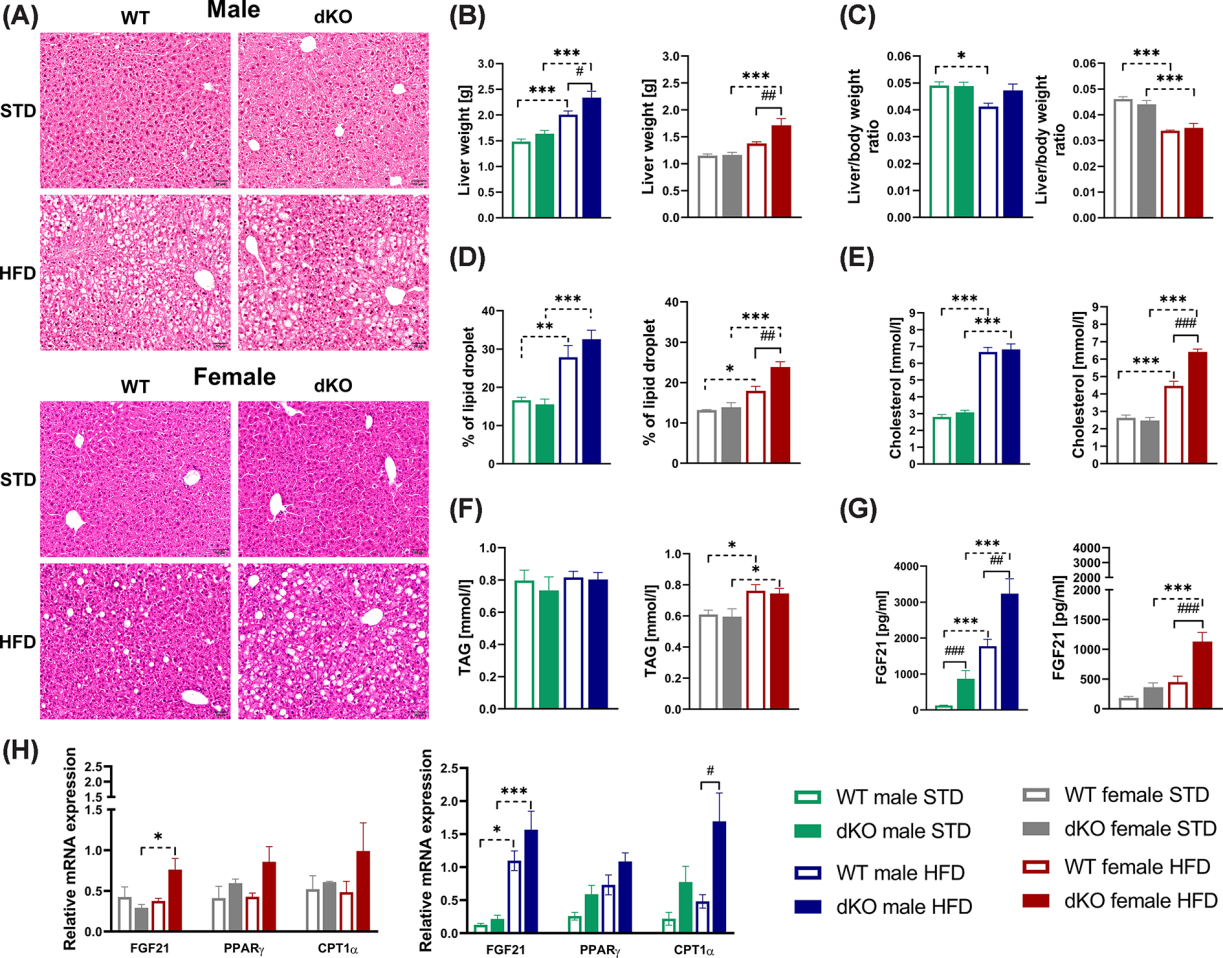
Effect of GPR10 and NPFFR2 deletion on liver steatosis and plasma cholesterol levels Hematoxylin/eosin staining of liver slices at a 20x magnification in representative photomicrographs of WT and dKO mice fed either STD or HFD (**A**). Liver weights in males and females (*n*=9–10) (**B**) and liver-to-body weight ratio in males and females (**C**). Liver histology quantification as a percentage (%) of lipid droplet in the liver area (*n*=7) in males and females (**D**). Cholesterol levels (*n*=5–8) in fasting plasma in males and females (**E**), fasting plasma TAG levels (*n*=9–10) (**F**), and free-fed mice plasma FGF21 levels (*n*=9–10) (**G**) in WT and dKO mice on either STD or HFD. Relative *Fgf21*, *Pparγ*, and *Cpt1α* gene expression in the livers of both males and females (*n*=3–5) (**H**). All data are expressed as the mean ± SEM and evaluated by two-way ANOVA with Bonferroni post hoc test. #*P*<0.05, ##*P*<0.01, and ###*P*<0.001 for WT vs. dKO mice on the same diet and *P<0.05, **P<0.01, and ***P<0.001 for STD vs. HFD mice of the same genotype.

The impact of deletion of GPR10 and NPFFR2 receptors on metabolic changes in the liver was studied. No significant differences were observed in liver weight between genotypes of both sexes when fed STD. However, both dKO males and females fed HFD, displayed significantly increased liver weight compared with their WT controls (Figure 4B). The liver-to-body weight ratio (Figure 4C) showed a significant decrease in mice of both genotypes and sexes when fed a HFD, except for dKO males fed HFD. These results were supported by liver histology showing differences in steatosis, manifested by enhanced accumulation of lipid droplets in males of both genotypes fed HFD (Figure 4A,D). Also, dKO females fed HFD exhibited a higher levels of lipid droplets in their livers (Figure 4A,D) and elevated cholesterol levels in fasting plasma (Figure 4E) compared with their WT controls.

The TAG levels did not change in the fasting plasma between the WT and dKO mice on STD, significant increase was observed only in females on HFD (Figure 4F). Both dKO males and females exhibited increased plasma levels of the hepatokine FGF21 when fed HFD (Figure 4G), but no significant genotype-dependent changes in *Fgf21* mRNA expression in the liver were observed (Figure 4H). When fed HFD, dKO males displayed a significant increase in *Cpt1α* (carnitine palmitoyltransferase 1*α*) gene expression levels in the liver compared with those in WT males (Figure 4H).

### Female dKO mice displayed increased anxiety-like behavior

**Figure 5:**
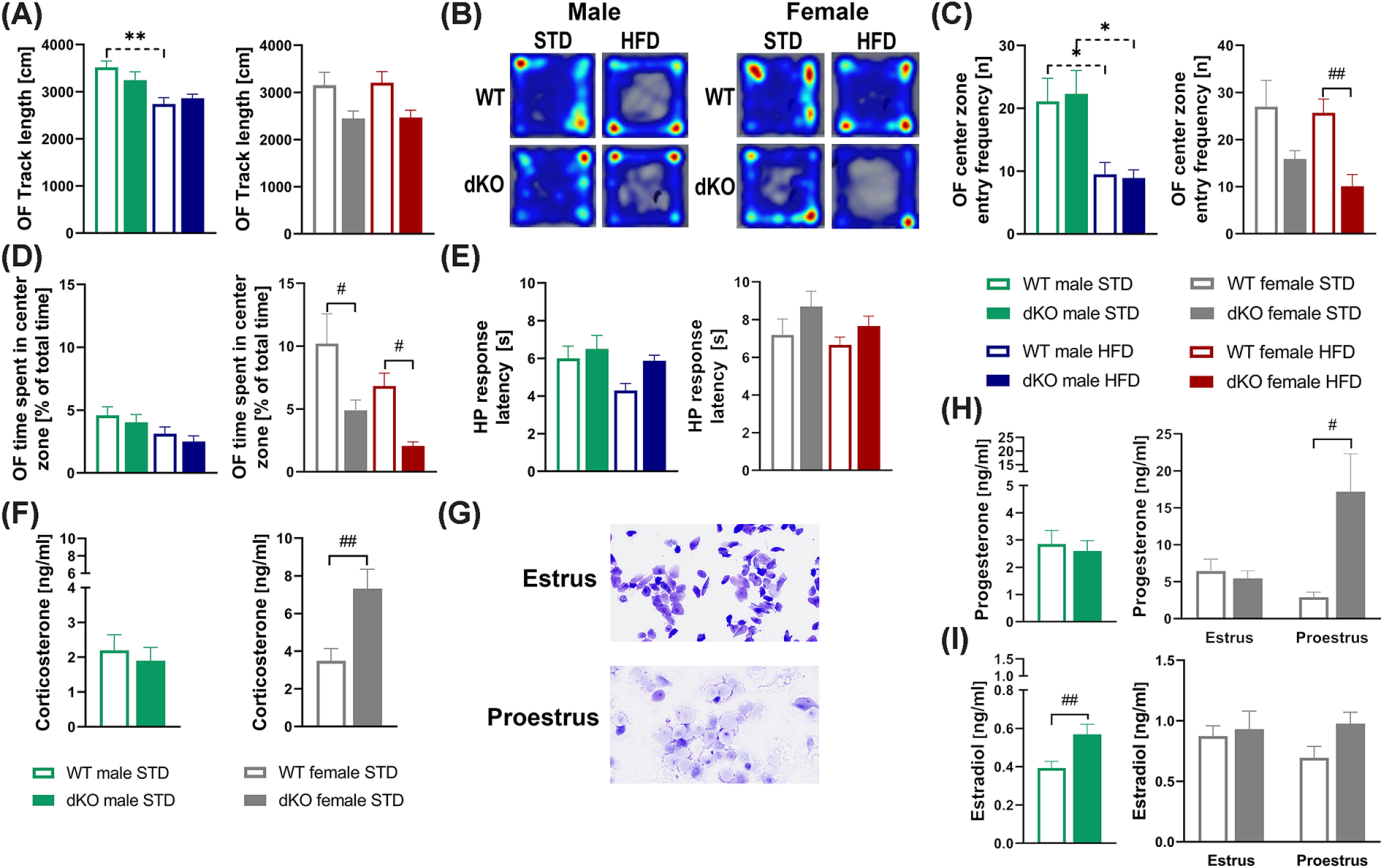
Behavioral experiments and hormonal levels in dKO mice Track length in the open field (OF) test for males and females (*n*=8–10) (**A**). Representative heat maps of WT and dKO mice show time spent at different locations within the OF for males and females fed either STD or HFD (**B**), the frequency of entries to the OF center zone for males and females (**C**), % of total time (10 min) spent in the center zone in OF for males and females (**D**). Latency to pain perception (paw licking) in the hot plate (HP) test for males and females (**E**). Corticosterone (**F**), progesterone (**H**), and estradiol (**I**) levels in free-fed plasma of WT and dKO mice on STD (*n*=8–10). Representative images of vaginal smears of the estrous cycle at a 200× magnification (**G**). Data are expressed as the mean ± SEM and were evaluated by two-way ANOVA with Bonferroni post hoc test (B**–**E) or by unpaired Student’s *t*-test (F–H). #*P*<0.05, ##*P*<0.01 for WT vs. dKO mice on the same diet and *P<0.05 and **P<0.01 for STD vs. HFD mice of the same genotype.

The effects of double deletion on behavior were subsequently determined using standard behavior tests. No significant alterations in traveled distance between genotypes of 21-week-old mice were observed in the OF test (Figure 5A). Both WT and dKO males fed HFD showed less exploratory behavior in the OF test than their respective WT controls (Figure 5B–D). In addition, dKO females spent less time and had fewer entries into the center zone, particularly those on HFD (Figure 5B–D). The HP test showed a tendency toward increased nociceptive response latency in dKO mice of both sexes compared with their respective WT controls (Figure 5E).

Female dKO mice showed significantly higher levels of plasma corticosterone than WT controls, but no increase was observed in males (Figure 5F). Since the estrous cycle can impact the response to threatening stimuli in behavioral tests assessing anxiety and fear, cytological evaluation of vaginal smears (Figure 5G) was performed. This revealed that there were 11 WT and 10 dKO females in estrus, 9 WT and 9 dKO females in proestrus, and 1 dKO female in diestrus. During the proestrus phase, dKO females exhibited significantly higher progesterone levels (Figure 5H), while the levels of estradiol remained similar to those of the WT controls (Figure 5I).

### dKO mice showed increased body weight on STD and no changes in energy expenditure

To investigate the effect of lack of GPR10 and NPFFR2 receptor on energy metabolism under standard and obesogenic conditions, we subjected dKO and WT mice into indirect calorimetry. Before the onset of HFD feeding, 18-week-old mice from Experiment 2 showed no changes in body weight between genotypes of mice on STD (Supplementary Figure S2A). EE displayed a pronounced circadian pattern corresponding to expected periods of activity and feeding during the night (Supplementary Figure S2C,E), and the multilinear regression did not reveal a significant impact of genotype on EE. The respiratory quotient (RQ), a marker of substrate oxidation, did not differ between genotypes (Supplementary Figure S2D). However, it did exhibit the expected circadian rhythm (Supplementary Figure S2F) of a lower RQ corresponding to higher oxidation of lipids during the day phase and a higher RQ corresponding to higher oxidation of carbohydrates during the night phase in all groups.

**Figure 6:**
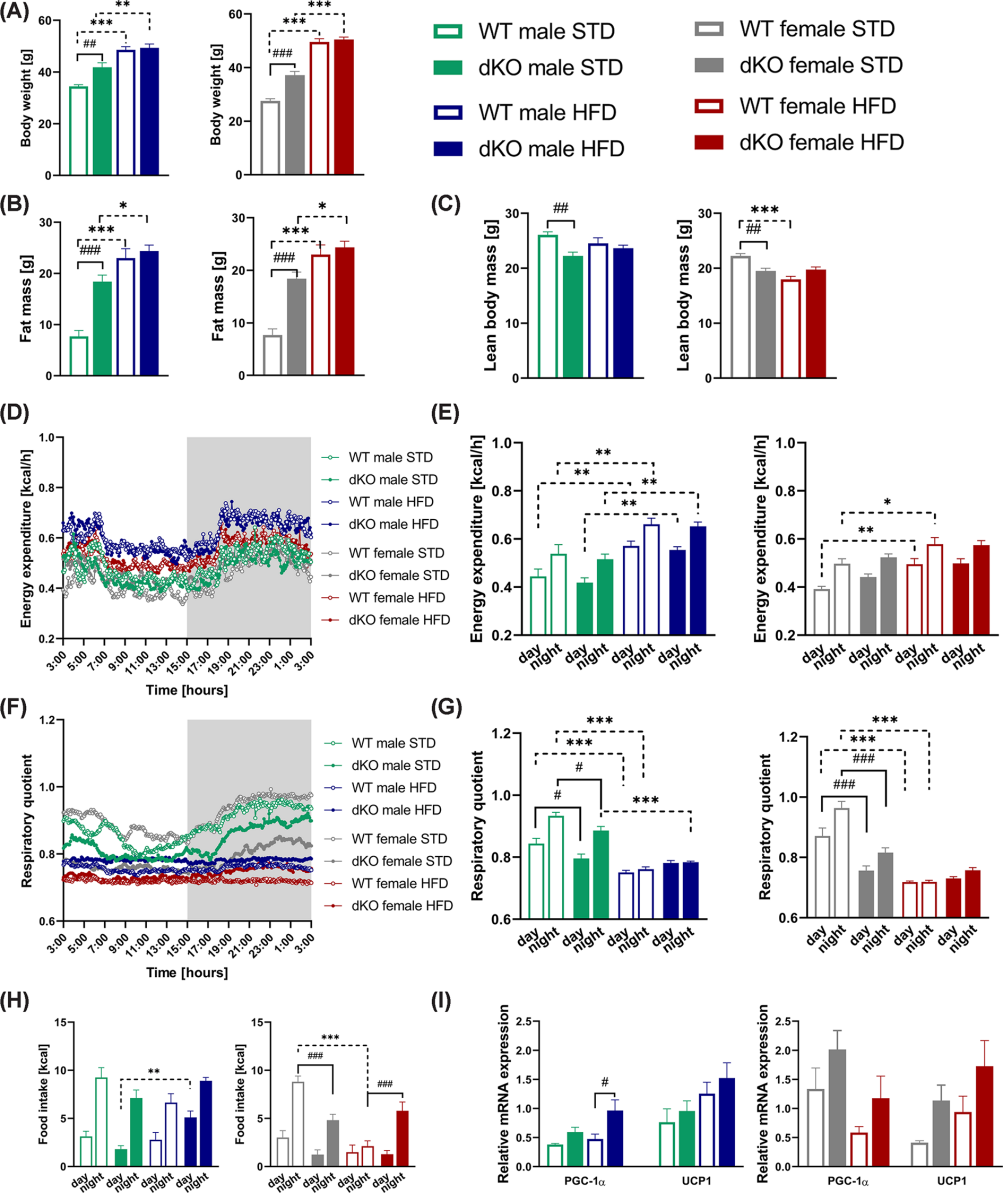
Characterization of whole-body energy homeostasis after 4 months of HFD feeding Energy metabolism of 34-week-old mice (*n*=9–10) was assessed for 48 h using indirect calorimetry and the last 24 h were used for analysis. Body weight before the measurement (**A**), fat mass measured by DEXA (**B**), lean mass measured by DEXA (**C**), time course of energy expenditure measurement [kcal/h] (**D**), energy expenditure during the light and dark phase of the day (**E**), time course of respiratory quotient (**F**), respiratory quotient during the light and dark phase of the day (**G**), and caloric food intake [kcal] (**H**). Relative expression of *Pgc-1α* and *Ucp1* in brown adipose tissue (*n*=5) (**I**). Data are expressed as mean ± SEM and determined by two-way ANOVA with Bonferroni post hoc test. #*P*<0.05, ##*P*<0.01 and ###*P*<0.001 for WT vs. dKO mice on the same diet and *P<0.05, **P<0.01, and ***P<0.001 for STD vs. HFD mice of the same genotype.

Four months of HFD feeding resulted in increased body weight in both males and females of both genotypes (Figure 6A). 34-week-old dKO mice of both sexes fed STD exhibited a significantly higher body weight than WT controls (Figure 6A). The DEXA scanner showed a significant increase in fat mass in dKO mice of both sexes fed STD (Figure 6B) and a decrease in lean body mass (Figure 6C). Energy metabolism of mice was assessed as mentioned above. The EE per animal subsequently differed as a function of different body weights (Figure 6D,E), but the multilinear regression did not reveal a significant impact of genotype on EE (see Methods). While a commonly used normalization of EE outcomes to body weight or lean body mass might bring misleading results [33], the suggested solution of such a complex situation is either to measure mice before the onset of obesity, to uncover any differences that could lead to obesity in older age, or to evaluate obtained data using ANCOVA or regression-based methods [33]. We have employed both approaches, and we have confirmed that no differences in EE that can be detected by our indirect calorimetry system are present between genotypes in either situation.

Lower RQ and circadian differences in RQ were observed in all HFD-fed mice of both genotypes and in dKO of both sexes fed STD, indicating higher oxidation of lipids than carbohydrates during both the day and night (Figure 6F,G). In females, the lower RQ might be caused by lower carbohydrate-rich food intake and the subsequent oxidation of lipids from body stores of dKO compared with WT STD-fed mice (Figure 6G,H). However, despite a similar food intake (Figure 6H), dKO males displayed a significantly lower RQ during both the day and night phase of the measurement compared with that of WT STD-fed mice (Figure 6G), suggesting a possible more general influence on increased lipid oxidation in dKO mice.

Moreover, the gene expression levels of *Pgc-1α* were significantly higher in dKO males fed HFD than in WT controls, but no changes in *Ucp1* gene expression levels were observed (Figure 6I).

### No alterations in PrRP and NPFF levels in dKO mice

**Figure 7:**
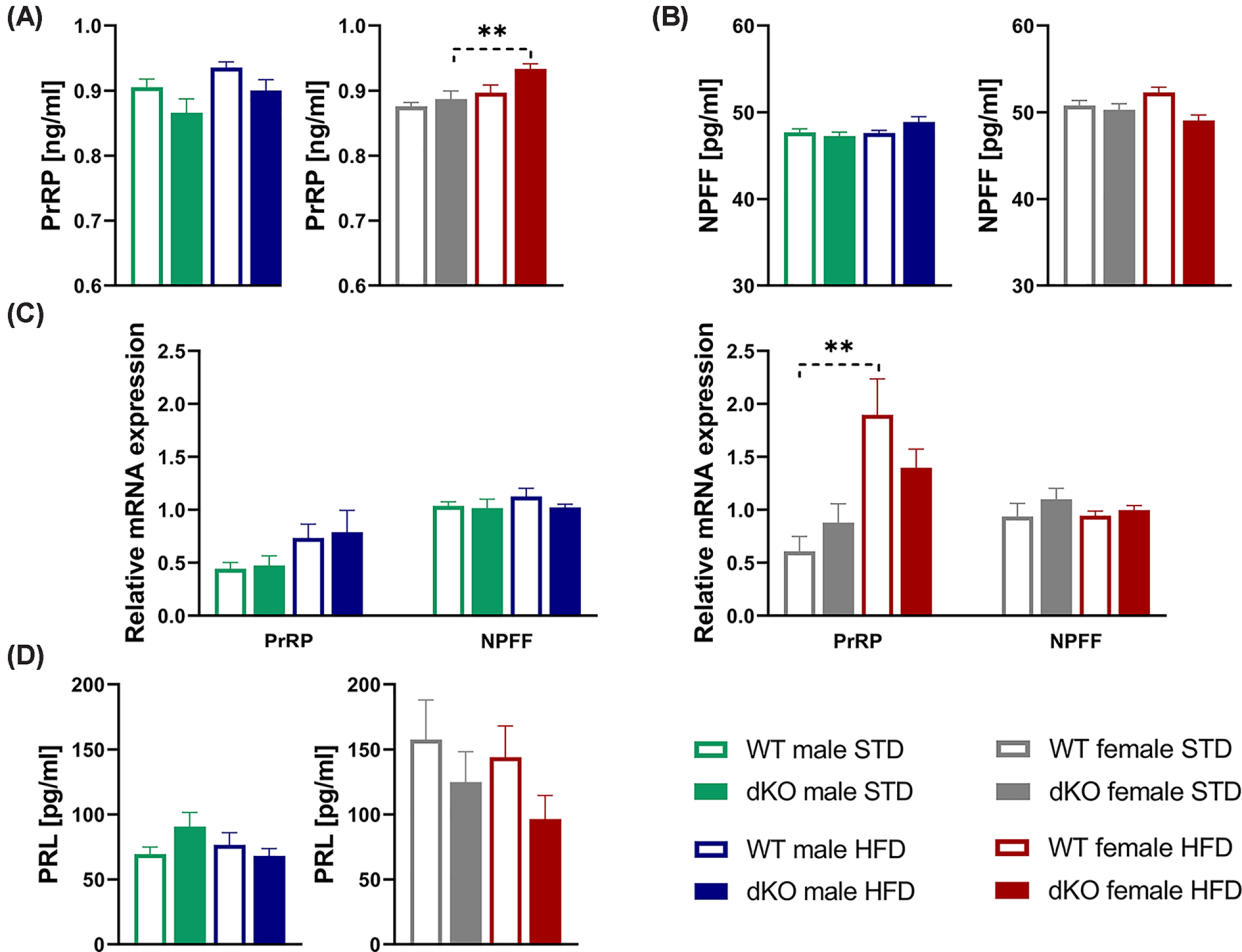
Levels of PrRP, NPFF, and prolactin in dKO mice and gene expression in hypothalamus Free-fed plasma PrRP levels (**A**) and NPFF levels (**B**) in WT and dKO mice on either STD or HFD (*n*=9–10). Relative mRNA expression of *Prrp* and *Npff* in the hypothalamus (*n*=5) (**C**). Free-fed plasma PRL levels in males and females (*n*=4–10) (**D**). Data are expressed as the mean ± SEM and evaluated by two-way ANOVA with Bonferroni post hoc test. **P<0.01 for STD vs. HFD mice of the same genotype.

No changes in PrRP and NPFF plasma levels (Figure 7A,B) or mRNA gene expression levels of *Prrp* and *Npff* in the hypothalamus were found between genotypes (Figure 7C). Moreover, dKO mice did not exhibit changes in prolactin plasma levels (Figure 7D).

## Discussion

Stable lipidized PrRP analogs, which activate both GPR10 and NPFFR2 receptors, showed significant appetite reduction and weight loss after peripheral administration in rodents with DIO [25,26]. This highlights the potential of dual agonists targeting GPR10 and NPFFR2 in weight loss therapy [26,34]. In previous studies, the effects of GPR10 deficiency and NPFFR2 deficiency on food intake and energy metabolism were investigated [17–21,35]. Here, for the first time, we aimed to characterize dKO mice to gain a better understanding of how GPR10/NPFFR2 deficiency influences the regulation of energy metabolism in both male and female mice. These investigations were conducted on the BL/6 mouse strain background, which is widely employed in metabolic disease research due to its susceptibility to DIO and insulin resistance [36]. We acknowledge the existence of differences between C57BL/6J and C57BL/6N mice, particularly in gene variations affecting coding sequences that may influence various physiological, biochemical, and neurobehavioral systems [37,38]. In DEXA lean mass analysis, two mouse centers observed increased fat mass in 6N mice compared with 6J, while another center did not detect any significant changes [37]. However, there were no significant variations in morphological features, including X-ray analysis of the skeleton. Free-fed and fasting plasma glucose levels were higher in 6N mice than in 6J mice by at least two centers. C57BL/6J mice have a mutation in the nicotinamide nucleotide transhydrogenase (*Nnt*) gene which is involved in β-cell mitochondrial metabolism, resulting in altered glucose homeostasis and insulin secretion [37,39]. On the other hand, C57BL/6N mice do not have this mutation. C57BL/6N mice on a HFD demonstrated increased plasma insulin and blood glucose, whereas these results were delayed in the C57BL/6J mice [40]. However, blood glucose levels can be influenced by animal handling, sample processing, and the use of anesthetics. These inconsistencies suggest operational differences between centers and laboratories.

Previous research indicates that the PrRP-NPFF system (or GPR10-NPFFR2, respectively) is involved in the regulation of glucose homeostasis. NPFF is involved in regulating glucose balance, and its deficiency in NPFF KO mice leads to improved glucose tolerance and reduced blood glucose levels compared with WT mice [41]. However, NPFFR2 KO mice exhibited impaired glucose tolerance and showed significantly greater glucose excursions in OGTT that were exacerbated by HFD [21]. PrRP KO mice displayed increased blood glucose and insulin levels [5,42]. Previously, spontaneous mutation in the *Gpr10* gene in Otsuka-Long-Evans Tokushima Fatty rats led to hyperglycemia, hyperinsulinemia, and insulin resistance [43]. Additionally, deficiency of GPR10 in mice led to an abnormal increase in insulin secretion in response to HFD [19]. In this study, feeding HFD to dKO mice resulted in reduced glucose tolerance, hyperinsulinemia, and insulin resistance. Clearly, the lack of GPR10-NPFFR2 signaling disturbed the regulation of glucose metabolism. Moreover, NPFFR2 KO males fed HFD previously exhibited increased levels of FGF21 in plasma [21]. FGF21 is known to play a role in regulating metabolism and promoting beneficial effects on glucose and lipid homeostasis [44]. The increased production of FGF21 in 25-week-old dKO mice fed HFD suggests a potential compensatory mechanism to alleviate metabolic stress, insulin resistance, and glucose dysregulation [44].

Previous studies demonstrated that GPR10 KO mice exhibited moderate obesity when fed STD as a result of higher adiposity accompanied by elevated plasma leptin levels [17–19]. In the present study, 25-week-old dKO males fed STD exhibited increases in final body weight, and active ghrelin levels, and deficiency of both receptors resulted in hyperphagia-induced increased body weight. Moreover, 34-week-old dKO mice of both sexes fed STD developed late-onset obesity. DEXA scan revealed an increase in fat mass, which was further confirmed by weighing excised fat mass. In contrast, 25-week-old dKO males fed HFD showed reductions in white adipose tissue mass and leptin levels, which may be caused by their elevated levels of estradiol. This pattern was similarly observed in NPFFR2 KO males, who displayed decreased body weight when fed HFD [21]. This suggests that GPR10-NPFFR2 signaling is involved in the regulation of the hypothalamic-pituitary-gonadal (HPG) axis and that a higher level of estradiol in dKO males fed HFD could improve glucose metabolism, adiposity, the distribution of body fat, and glucose metabolism. In contrast, dKO females exhibited obesity, increased white adipose tissue mass, elevated plasma cholesterol levels and the accumulation of lipids in the liver when they were fed HFD. Their elevated corticosterone level indicated a disturbed HPA axis that probably contributes to this, as elevated corticosterone is associated with insulin resistance, impaired glucose utilization, increased fat mass leading to hepatic steatosis, and metabolic syndrome [45].

In the present study, dKO females showed increased anxiety-like behavior in the OF test. Previously, corticosterone levels were found to be increased in GPR10 KO females, while no difference was observed in GPR10 KO males versus their WT controls [17]. These findings were also observed by our group, although they have not been published. However, no alterations in the behavior were observed in GPR10 KO [19] nor in NPFFR2 KO mice [21]. Lin et al. demonstrated that NPFFR2 KO mice exhibited impaired HPA negative feedback and reduced anxiety-like behaviors following exposure to single prolonged stress [46]. But depressive- and anxiety-like behavior were not different between WT control mice and NPFFR2 KO mice [46]. This indicates that increased anxiety-like behavior and corticosterone levels in dKO females could be caused by the deletion of the GPR10 receptor rather than the NPFFR2 receptor. A recent study found that the mutation-induced loss of GPR10 function can result in weight gain in humans followed by the development of anxiety [35]. Thus, targeting GPR10 and NPFFR2 receptors may be beneficial not only for obesity treatment but also for attenuating anxiety since the deficiency of both receptors resulted in anxiety-like behavior in dKO females.

In addition, the energy metabolism of mice was investigated at the age of 34 weeks. The EE per animal subsequently differed as a function of different body weights, but the multilinear regression did not reveal a significant impact of genotype on EE. Our results do not fully correspond to previously published studies, probably due to different methodological approaches for EE normalization. Lowered EE was detected in single GPR10 KO animals when inappropriate normalization per kg of body weight was used [17], and conflicting results were obtained using normalization of EE per animal [19,35]. In NPFFR2 KO mice, lowered EE was detected only in mice fed HFD, but not STD [20]. In the present study, EE was normalized to metabolically active tissue (lean body mass + [0.2] × fat mass), but as the contribution of fat mass to EE has not been clearly set yet [47,48], this type of normalization may distort data obtained from mice differing in their body weights. Thus, the reported reduction of EE in GPR10 KO or NPFFR2 KO models might be influenced by normalization of original data.

Both sexes of dKO mice fed STD exhibited a shift toward a lower RQ, suggesting increased lipid oxidation. In females, this shift might partially be explained by the lower food intake of dKO mice, leading to higher oxidation of lipid body stores. However, this difference was also significant in males, where no significant changes in food intake were detected. The shift toward lipid oxidation may be either a cause or a consequence of the increased body weight of STD-fed dKO males and females. As only a nonsignificant trend toward a lower RQ in dKO males and females was observed at the start of the experiment at the age of 18 weeks, when mice did not differ in body weights, we cannot decide which of the options, i.e. cause vs. consequence, is true.

There is evidence of sex differences in the hypothalamic regulation of homeostasis and feeding behaviors [49]. In humans, these differences are apparent in various physiological characteristics, such as fat distribution and metabolic health. For example, adult females generally have a higher fat mass, lower lean body mass, and tend to deposit fat subcutaneously, whereas males typically have more visceral fat. While the clinical and epidemiological patterns of these differences are well established, their biological basis remains unclear. Studies in mice suggest that adipose tissue mass and gene expression are regulated by sex-specific gene networks, including inflammatory and developmental genes some of which influenced by sex steroid hormones [50,51].

In conclusion, dual agonists targeting GPR10 and NPFFR2 show potential in weight loss therapy. Clearly, the impact on glucose metabolism, adiposity, and lipid accumulation varied between male and female dKO mice, highlighting the significance of sex-specific approaches in obesity research. This study provided insights into metabolic regulation associated with disrupted GPR10-NPFFR2 signaling, contributing to the development of potential therapeutic interventions for obesity-related issues.

## Supplementary Material

Supplementary Figures S1-S2 and Table S1

## Data Availability

All relevant data are included in the manuscript. The raw data supporting the study’s findings are available from the corresponding author upon request.

## Abbreviations

AUC: area under the curve
DEXA: dual-energy X-ray absorptiometry
dKO: double knockout
DMN: dorsomedial hypothalamic nucleus
EE: energy expenditure
GPR10: G-protein coupled receptor 10
HFD: high-fat diet
NPFFR2: neuropeptide FF receptor 2
NTS: nucleus tractus solitarius
OGTT: oral glucose tolerance test
PrRP: prolactin-releasing peptide
VHM: ventromedial hypothalamus
