# Supplementary material for: Deficiency of GPR10 and NPFFR2 receptors leads to sex-specific prediabetic syndrome and late-onset obesity in mice
Source: Biosci Rep. 2024 Oct 23;44(10):BSR20241103. doi: 10.1042/BSR20241103 (PMC11499387; doi:10.1042/BSR20241103)
Supplement: Supplementary Figures S1-S2 and Table S1 [file BSR-2024-1103_supp.pdf]

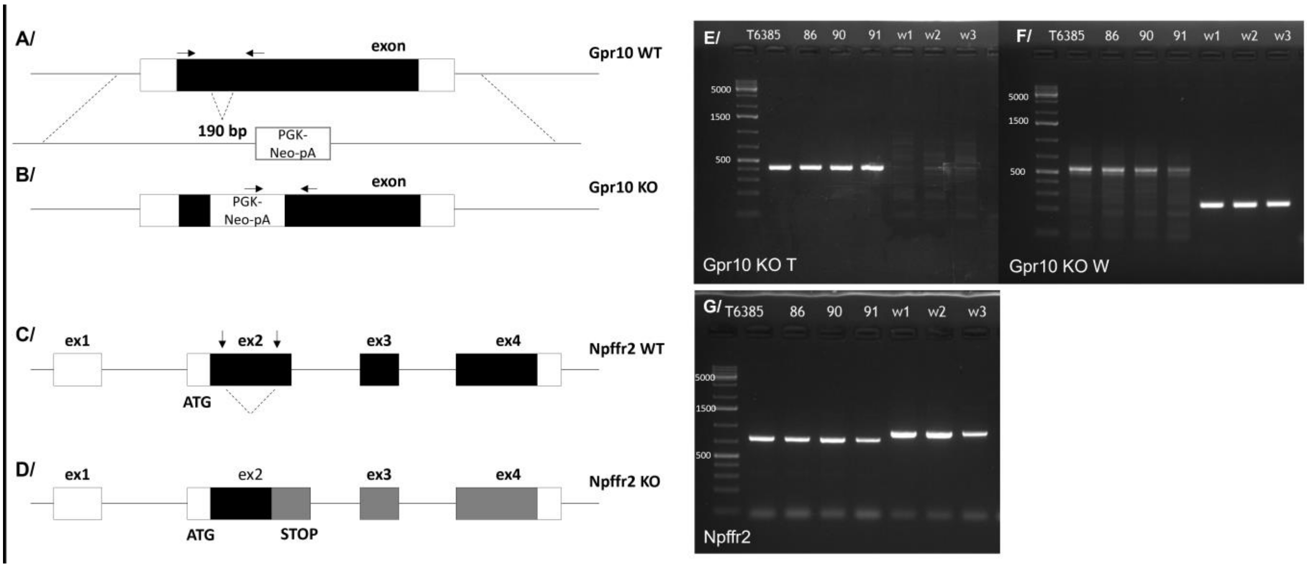

Supplementary Figure 1: Deletion in *Gpr10* and *Npffr2* coding sequence. Schematic diagram of the mouse Gpr10 (**A**) and Npffr2 (**C**) gene and the modified region (**B**) and (**D**) in KO mice. Gel showing deletion in dKO animals T6385, 86, 90 and 91 and wild-type (WT) mice W1, 2, 3 using primers *Gpr10* T and W (**E, F**) and *Npffr2*-F and R (**G**).

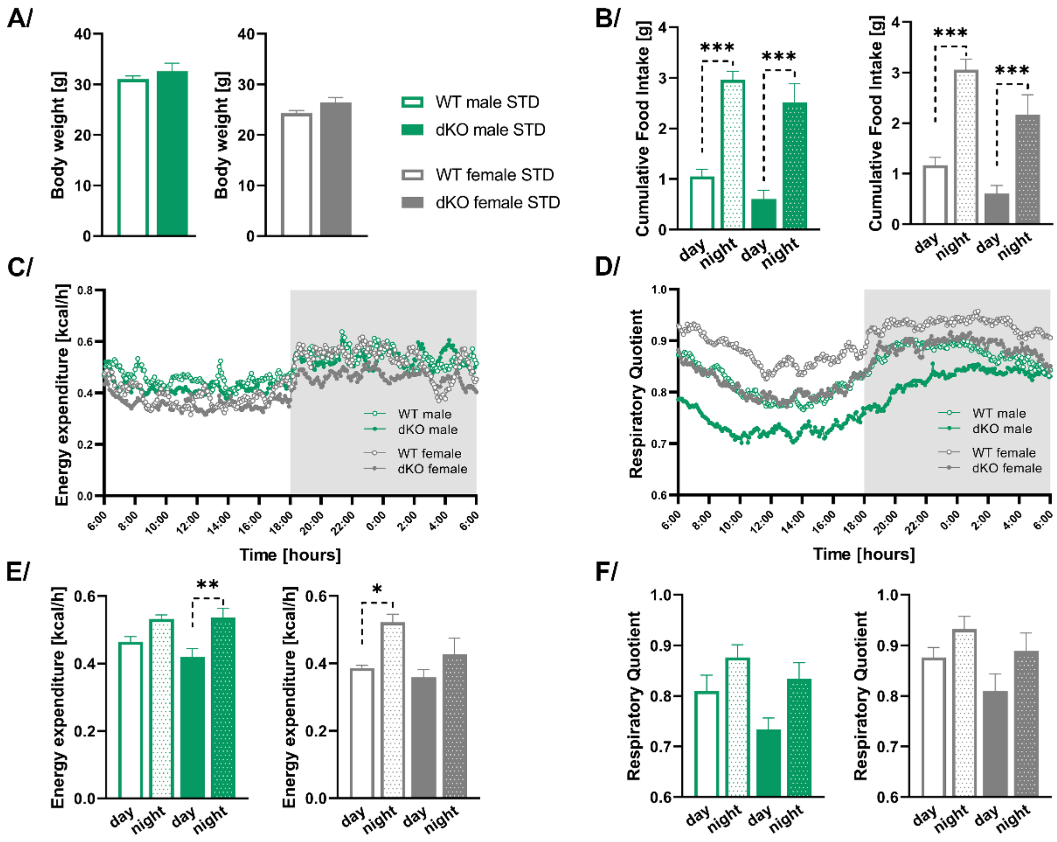

Supplementary Figure 2: Characterization of energy expenditure before the onset of HFD feeding. Energy metabolism of 18-week-old mice (n = 9-10) on STD was assessed for 48 hours using indirect calorimetry and the last 24 hours were used for analysis. Body weight before the measurement (**A**), cumulative food intake during the light and dark phase of the day (**B**), time course of energy expenditure measurement (**C**), time course of respiratory quotient (**D**), energy expenditure during the light and dark phase of the day (**E**), respiratory quotient during the light and dark phase of the day (**F**). The points in (**B, C**) represent individual measurements over time. Data are expressed as mean ± SEM (n=6-10) and were analyzed by unpaired t-test (**A**) or two-way ANOVA with Bonferroni post hoc test (**B**, **E**, **F**). *p < 0.05, **p < 0.01, and ***p < 0.001 for STD vs. HFD mice of the same genotype.

Supplementary Table 1. Sequences of primers.

Gene Name	Gene ID	Forward Primer	Reverse Primer	Abbreviation
Cpt1a	12894	GCAGCTCGCACATTACAAGGACAT	AGCCCCCGCCACAGGACACATAGT	Carnitine palmitoyltransferase 1a
Fgf21	56636	AGCGCAGCCCTGATGGAATGGAT	CTGGAGGAGGGGGCTGGAGTC	Fibroblast growth factor 21
Gapdh	14433	CCCGGCATCGAAGGTGGAAGAGT	CTGACGTGCCGCCTGGAGAAAC	Glyceraldehyde-3-phosphate dehydrogenase
Npff	54615	AGAACTGTCTGGCCTGCGGAT	TTCAGCCCCAGAGGTTTGGCAG	Neuropeptide FF-amide peptide precursor
Pgc1a	19017	CCCAAAGGATGCGCTCTCGTT	TGCGGTGTCTGTAGTGGCTTGATT	Peroxisome proliferative activated receptor gamma-coactivator 1 alpha
Pparg	19016	GCCTTGCTGTGGGGATGTCTC	CTCGCCTTGGCTTTGGTCAG	Peroxisome proliferative activated receptor-gamma
PrRP	226278	CCAGGTCCGGTGACATCCCT	CTGCCTGGTACACGGGTCGT	Prolactin releasing hormone
Ucp1	22227	CACGGGGACCTACAATGCTTACAG	CACGGGGACCTACAATGCTTACAG	Uncoupling protein 1
Ywhaz	22631	CCTCGGCCAAGTAACGGTAGT	CGGAGCTGCGTGACATCTG	Tyrosine 3-monooxygenase/tryptophan 5-monooxygenase activation protein zeta
